# The spatiotemporal characteristics of influenza A and B in the WHO European Region: can one define influenza transmission zones in Europe?

**DOI:** 10.2807/1560-7917.ES.2017.22.35.30606

**Published:** 2017-08-31

**Authors:** Saverio Caini, Wladimir J Alonso, Clotilde El-Guerche Séblain, François Schellevis, John Paget

**Affiliations:** 1 Netherlands Institute for Health Services Research (NIVEL), Utrecht, The Netherlands; 2 Origem Scientifica, São Paulo, Brasil; 3 Sanofi Pasteur, Lyon, France; 4 Department of General Practice and Elderly Care Medicine, EMGO Institute for Health and Care research, VU University Medical Center, Amsterdam, The Netherlands

**Keywords:** Influenza, influenza type A, influenza type B, surveillance systems, timing of epidemics, influenza transmission zones

## Abstract

We aimed to assess the epidemiology and spatiotemporal patterns of influenza in the World Health Organization (WHO) European Region and evaluate the validity of partitioning the Region into five influenza transmission zones (ITZs) as proposed by the WHO. We used the FluNet database and included over 650,000 influenza cases from 2000 to 2015. We analysed the data by country and season (from July to the following June). We calculated the median proportion of cases caused by each virus type in a season, compared the timing of the primary peak between countries and used a range of cluster analysis methods to assess the degree of overlap between the WHO-defined and data-driven ITZs. Influenza A and B caused, respectively, a median of 83% and 17% cases in a season. There was a significant west-to-east and non-significant (p = 0.10) south-to-north gradient in the timing of influenza activity. Typically, influenza peaked in February and March; influenza A earlier than influenza B. Most countries in the WHO European Region would fit into two ITZs: ‘Western Europe’ and ‘Eastern Europe’; countries bordering Asia may be better placed into extra-European ITZs. Our findings have implications for the presentation of surveillance data and prevention and control measures in this large WHO Region.

## Introduction

The World Health Organization (WHO) European Region includes 53 countries covering a total population of nearly 900 million inhabitants. Influenza has a substantial medical and economic burden every season in the World Health Organization European Region (WHO/Europe) [1-4], and the reduction of influenza-related morbidity and mortality has long been recognised as a priority health objective in Europe.

Influenza viruses spread rapidly and their transmission can be favoured by anthropogenic factors such as the increase in international travel and commuters’ mobility [5-8]. The WHO European Region has become increasingly interconnected, especially since the end of the Cold War in 1991 and the eastward enlargement of the European Union (EU), and it is widely accepted that efficient and timely influenza surveillance must be coordinated at national and supranational level. Countries in the west of Europe have been sharing epidemiological and virological data via the European Influenza Surveillance Scheme (EISS) since 1996 [9,10], and this collaborative project became in 2008 the European Influenza Surveillance Network (EISN) coordinated by the European Centre for Disease Prevention and Control (ECDC). The WHO Regional Office for Europe extended the surveillance activities of EISS to all countries of the WHO European Region in 2008 [10].

Influenza epidemics typically peak during the northern hemisphere winter (November to March) in the WHO European Region [11,12]. Earlier research found that the timing of influenza activity moves across Europe, frequently travelling from west to east and, less frequently, from south to north [13,14], suggesting that there may be some heterogeneity in the timing of influenza epidemics among countries of the WHO European Region.

**Figure 1:**
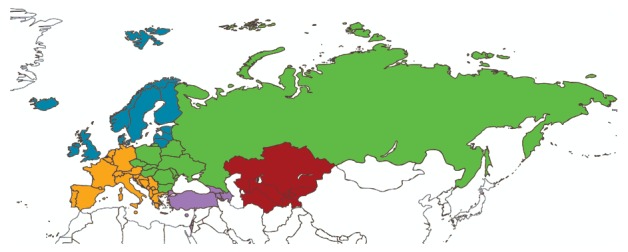
Countries included in the WHO European Region and their subdivision into the WHO Influenza Transmission Zones

The WHO influenza transmission zones (ITZs) were established by the WHO Global Influenza Programme during the 2009 pandemic (personal communication, Julia Fitzner, WHO, April 2017). The 53 countries of the WHO European Region fall into five different ITZs: Northern Europe (10 countries), South West Europe (22 countries), Eastern Europe (10 countries), Western Asia (six countries) and Central Asia (five countries) (Figure 1).

WHO: World health Organization.

Based on [15]. Blue: Northern Europe; orange: South West Europe; green: Eastern Europe; red: Central Asia; violet: Western Asia.

The ITZs were defined as large supranational areas encompassing “countries, areas or territories with similar influenza transmission patterns” [15]. As far as we know, no study has been conducted to verify whether the partitioning of the WHO European Region is justified from an epidemiological and/or virological standpoint. The aim of the present study was therefore to assess the epidemiology and spatiotemporal patterns of influenza A and B in the WHO European Region, and evaluate the validity of its partitioning into five ITZs as proposed by WHO.

## Methods

### Source of data

FluNet is a publicly available, web-based database maintained by the WHO Global Influenza Surveillance and Response System since 1995, in which National Influenza Centres (NICs) from countries around the world enter epidemiological and virological data on influenza on a weekly basis [16]. On 12 October 2015, we downloaded the weekly number of laboratory-confirmed influenza cases reported to the national surveillance systems of all countries in the WHO European Region from week 1/1999 onwards, broken down by virus type (influenza A, B), subtype (H1N1, H1N1pdm09, H3N2, not subtyped) and lineage (Victoria, Yamagata, not characterised).

Because seasonal influenza epidemics typically occur in the winter months in temperate countries of the northern hemisphere, we opted to use ‘country season’ as the unit of analysis, defined as the period between 1 July of a year and 30 June of the following year in a given country. Therefore, we finally included in the analyses the data from week 27/1999 to week 26/2015.

### Descriptive analysis

For each country and season, we determined the proportion of laboratory-confirmed influenza cases that were caused by each virus type, subtype and lineage, and calculated the corresponding median value for all countries in the WHO European region. We then calculated the percentage of country seasons in which a given virus type, subtype and lineage accounted for 50% or more of all reported influenza cases. In order to increase the reliability of the results, country seasons with fewer than 100 overall reported influenza cases were excluded from this analysis. This number was chosen as a trade-off between the necessity to have a sufficiently high number of cases to estimate key epidemic parameters (including the timing of the epidemic peak), and the requirement to include as many countries as possible, which was important given the main objective of our analysis. 

### Spatiotemporal patterns of influenza epidemics

Data before 2009 were not used for the study of spatiotemporal patterns of influenza epidemics in the WHO European Region because they were not complete for most countries. The pandemic season 2009/10 was also excluded as it was an atypical season with the introduction of a novel pandemic strain (influenza A(H1N1)pdm09) [17], hence not suitable for seasonal analyses. Spatiotemporal analyses were therefore performed from week 27/2010 to week 26/2015.

For each site, we first de-trended the time series with a quadratic polynomial. We then generated a periodic annual function (PAF) of each time series by summing up the annual, semi-annual and quarterly harmonics as obtained by Fourier decomposition [18,19]. The timing of the primary peaks of the PAF was extracted and compared between sites, as based on their latitude (defined as the latitude of the NIC; in countries with more than one NIC, we chose the one situated in the largest city). The timing of the primary peak indicates the period when the maximum intensity of disease burden usually takes place. Primary peaks were also extracted separately for influenza A and B.

### Influenza transmission zones

We chose a cluster analysis approach to obtain data-driven ITZs using the country season as the unit of analysis. Several algorithms are available to group objects into a set of mutually exclusive and exhaustive clusters. Here, there was no a priori reason to prefer any specific cluster analysis technique; therefore, we selected a procedure whereby the outputs of several cluster models were compared with one another in order to identify groups of countries that were consistently (i.e. across different models) assigned to the same cluster. We used a multiple cluster approach to draw robust conclusions and not be dependent on a single clustering methodology or a set of inputted parameters.

A common requirement for most cluster analysis algorithms is that there must not be any missing values for the variables that are used for the analysis. In our analysis, this implies that all included countries must have influenza surveillance data for all seasons. Because of this requirement, we limited the database used for the cluster models to data from four consecutive seasons (2011/12 to 2014/15) in 37 countries (see below for details). This selection was made as there was a substantial reduction in the number of countries (from 37 to 23) when extending the database to 2010/11, and no gain by reducing the number of seasons to three (by dropping the 2011/12 season).

In each season and country, we calculated the start and the peak of the influenza season, defined retrospectively as the first week in which at least 10% of all reported cases had occurred [20], and, respectively, the week with the highest number of reported cases. As the epidemics caused by the different influenza virus types and subtypes may differ in timing in the same country and season, the week of start and peak were calculated for all influenza cases taken together and separately for influenza A(H1N1)pdm09, A(H3N2) and B (if there were fewer than 100 cases for a given virus (sub)type, the start and peak of the epidemic caused by that virus (sub)type were assumed to coincide with those of the overall influenza season). We then calculated the median start and peak week of the influenza season (overall and by virus (sub)type) across seasons.

We fitted several cluster analysis models by varying the statistical method and the variables inputted in the model. In terms of the clustering algorithm, we used complete linkage, average linkage and k-means clustering. Concerning the model parameters, we hypothesised that the ITZs may differ with regard to the timing of the influenza season, the influenza virus mixing by season, or both. Accordingly, we initially fitted cluster models that included ‘timing’ parameters only (season-specific or median start and/or peak of the influenza season, for all influenza cases or by virus (sub)type), ‘virus mixing’ parameters only (percentage of influenza cases caused by each virus (sub)type), or both sets of parameters. We present here results generated by the models that included the timing parameters only, as the other models did not yield consistent and epidemiologically meaningful results (i.e. the geographical clusters were too diverse or varied).

Overall, the cluster analysis was repeated 18 times using different models. As there was no a priori criterion to prefer any single model over the others, we opted to summarise the results by calculating, for each pair of countries, the proportion of the 18 cluster models in which both countries fell into the same cluster (‘proportion of agreement’) and used the following algorithm to identify data-driven ITZs.

#### Definition of the core cluster of countries in an influenza transmission zone

A core cluster was defined when it included at least three countries and was identified according to two criteria: (i) The first (or ‘internal’) criterion states that all core countries of an ITZ must have a proportion of agreement of 80–100% between one country and another. This criterion ensures that all countries in the ITZ fit in the same cluster. (ii) The second (or ‘external’) criterion states that all core countries of an ITZ must have a proportion of agreement < 70% with all countries belonging to a different ITZ. This criterion ensures that none of the countries in the ITZ fit into another ITZ. Together, these two criteria ensure that the ITZs are mutually exclusive, i.e. they rule out the possibility that a country may belong to more than one ITZ. Importantly, the separation of clusters was enhanced by our decision to impose a 10% buffer between the inclusion (≥ 80%) and the exclusion (< 70%) criterion.

#### **Expansion of existing influenza transmission zones by adding non-core countries**

The attribution of the remaining countries to an existing ITZ was made according to a relaxed version of the two criteria above. Namely, each remaining country was assigned to an existing ITZ if its proportion of agreement was 70–100% with all countries in that ITZ, and < 70% with all countries in a different ITZ.

#### **Countries not allocated to an influenza transmission zone**

All of the remaining countries were considered not allocated to any ITZ.

As the countries in the WHO European Region fall into five ITZs according to the WHO, we initially set the number of clusters in the models to five. As the results were not satisfactory (see below), we then modified the model’s settings by progressively reducing the number of clusters to four, three and two.

### Statistical software

The EPIPOI software [19] was used to study the spatiotemporal patterns of influenza epidemics. We used Stata version 14 (Stata Corp, College Station, United States) to conduct the cluster analysis. Maps were prepared using freely available software (http://mapchart.net/).

## Results

The FluNet database included 654,952 viral isolates collected during the study period (week 27/1999 through week 26/2015) in 47 countries of the WHO European Region, of which 80.1% were influenza A and 19.9% were influenza B. No influenza surveillance data were available in the FluNet database for six countries: Andorra, Cyprus, Monaco, San Marino, Tajikistan, and Turkmenistan. Samples were unevenly distributed across countries, seasons and (sub)type. Samples were predominantly (82.9%) from the period 2009 to 2015, with 24.1% from the pandemic season (2009/10) alone. Most samples (51.1%) were from five countries (France, Norway, Russian Federation, Sweden and the United Kingdom (UK)), which contributed more than 45,000 samples each.

### Descriptive analysis

There were 417 seasons from 43 countries with at least 100 reported influenza cases, ranging from a maximum of 16 seasons in Finland, France, Germany, Israel, Italy, Latvia, Norway, Portugal and the UK, to one season in Kyrgyzstan and Uzbekistan. There were no seasons with 100 or more reported influenza cases in four countries (Armenia, Azerbaijan, the former Yugoslav Republic of Macedonia and Montenegro), which were therefore excluded from further analyses. Influenza A and B accounted for a median 82.6% and 17.4% of cases in a season, respectively. Influenza A accounted for 50% or more of all reported influenza cases in 361 of 417 seasons (86.6% of all seasons), while influenza B predominated in 56 seasons (13.4%). In more than one third (36.6%) of the influenza A seasons, influenza A(H3N2) was dominant, followed by influenza A(H1N1)pdm09 (28.8% of the influenza A seasons), not subtyped influenza A (28.8%) and pre-pandemic influenza A(H1N1) (5.8%). Only 12.1% of reported influenza B cases were characterised, making it impossible to perform an analysis by lineage. In most seasons, different virus (sub)types were dominant in different countries: the only exceptions were the seasons 1999/00 and 2003/04, which were dominated by the A(H3N2) subtype across the whole Region, and the season 2009/10, where the pandemic strain caused more than 85% of influenza cases in all countries. The number of influenza samples (by virus type, subtype and lineage) reported in each country is available in Table 1.

**Table 1:** Laboratory-confirmed influenza samples reported in each country by virus type, subtype and lineage, FluNet database, WHO European Region, 1999–2015 (n = 649,620)

Country	Number of seasons ^a^	Influenza cases	A(H3N2)	Pandemic A(H1N1)pdm09	Pre-pandemic A(H1N1)	A not subtyped	B Victoria	B Yamagata	B not characterised	Median % A (min, max) ^b^	Median % B (min, max) ^b^
Albania	3	421	183	178	0	11	0	0	49	NA	NA
Austria	10	7,384	1,673	1,546	64	2,437	0	0	1,664	91.4 (42.5–100)	8.6 (0–57.5)
Belarus	5	2,653	462	1,279	0	315	0	11	586	79.0 (65.0–92.0)	21.0 (8.0–35.0)
Belgium	13	8,525	1,837	2,201	393	2,615	20	384	1,075	87.6 (45.2–100)	12.4 (0–54.8)
Bosnia and Herzegovina	2	681	57	477	0	136	0	0	11	NA	NA
Bulgaria	6	2,159	514	1,154	9	43	0	54	385	86.6 (12.8–100)	13.4 (0–87.2)
Croatia	12	8,086	1,561	3,438	79	2,217	0	0	791	88.9 (63.4–100)	11.1 (0–36.6)
Czech Republic	12	4,972	909	1,698	223	1,181	7	2	952	79.2 (17.0–99.9)	20.8 (0.1–83.0)
Denmark	10	11,598	2,261	3,760	52	2,880	400	768	1,477	82.6 (19.0–100)	17.4 (0–81.0)
Estonia	8	5,011	430	1,646	137	1,942	0	25	831	88.8 (54.8–100)	11.2 (0–45.2)
Finland	16	5,776	1,189	876	284	2,486	45	44	852	88.5 (26.2–100)	11.5 (0–73.8)
France	16	86,328	8,562	29,874	389	32,220	380	1,115	13,788	80.0 (45.5–99.9)	20.0 (0.1–54.5)
Georgia	6	2,724	271	1,802	1	14	0	0	636	80.6 (16.2–99.4)	19.4 (0.6–83.8)
Germany	16	23,002	9,865	4,231	2,142	2,421	1,144	1,215	1,984	83.8 (2.2–99.8)	16.2 (0.2–97.8)
Greece	11	18,058	1,644	14,489	259	97	197	85	1,287	86.7 (22.0–99.9)	13.3 (0.1–78.0)
Hungary	6	4,176	950	2,546	0	129	7	79	465	85.0 (48.8–100)	15.0 (0–51.2)
Iceland	6	1,086	490	252	0	61	0	0	283	83.0 (18.8–100)	17.0 (0–81.2)
Ireland	9	12,764	3,433	6,621	85	541	0	0	2,084	76.7 (38.8–99.9)	23.3 (0.1–61.2)
Israel	16	15,131	1,856	8,658	387	2,459	4	0	1,767	78.4 (13.2–99.9)	21.6 (0.1–86.8)
Italy	16	24,176	5,180	13,878	412	1,733	9	180	2,784	93.8 (17.1–99.6)	6.2 (0.4–82.9)
Kazakhstan	6	2,491	1,233	454	11	208	0	5	580	74.9 (59.0–97.2))	25.1 (2.8–41.0)
Kyrgyzstan	1	256	0	207	0	45	0	0	4	NA	NA
Latvia	16	15,304	2,233	1,812	519	7,046	35	161	3,498	87.7 (34.0–100)	12.3 (0–66.0)
Lithuania	5	2,677	415	1,464	1	363	0	39	395	77.6 (69.8–100)	22.4 (0–30.2)
Luxembourg	8	3,838	507	1,762	59	593	0	93	824	78.6 (49.3–99.7)	21.4 (0.3–50.7)
Malta	2	478	1	162	0	184	0	0	131	NA	NA
Republic of Moldova	5	3,281	230	2,681	0	1	0	6	363	72.8 (52.2–100)	27.2 (0–47.8)
The Netherlands	8	11,514	3,697	4,132	14	1,678	152	975	866	88.8 (59.1–99.7)	11.2 (0.3–40.9)
Norway	16	64,054	4,260	14,392	454	27,362	621	2,202	14,763	78.9 (30.3–99.4)	21.1 (0.6–69.7)
Poland	6	6,433	103	3,912	12	1,611	11	0	784	84.3 (63.6–98.7)	15.7 (1.3–36.4)
Portugal	16	8,558	2,451	3,057	290	973	17	608	1,162	89.2 (2.2–100)	10.8 (0–97.8)
Romania	14	10,766	1,764	6,751	579	32	26	3	1,611	71.6 (13.1–100)	28.4 (0–86.9)
Russian Federation	15	82,364	20,143	39,837	2,979	2,952	5	29	16,419	78.2 (3.2–93.2)	21.8 (6.8–96.8)
Serbia	6	2,053	434	1,326	0	28	0	1	264	92.7 (52.9–100)	7.3 (0–47.1)
Slovakia	6	2,237	211	1,228	28	260	44	239	227	70.2 (45.4–100)	29.8 (0–54.6)
Slovenia	13	5,919	1,763	2,177	232	448	383	579	337	93.7 (16.0–99.6)	6.3 (0.4–84.0)
Spain	12	42,830	10,106	15,688	518	7,360	4	26	9,128	81.7 (24.8–100)	18.3 (0–75.2)
Sweden	14	54,336	4,613	18,029	80	20,156	282	380	10,796	90.8 (36.1–99.7)	9.2 (0.3–63.9)
Switzerland	15	17,588	1,969	3,456	234	7,680	652	465	3,132	77.8 (18.7–99.8)	22.2 (0.2–81.3)
Turkey	9	14,294	2,004	9,673	3	166	97	6	2,345	72.4 (29.9–99.9)	27.6 (0.1–70.1)
Ukraine	8	6,007	711	2,366	50	1,290	2	32	1,556	76.5 (35.7–99.0)	23.5 (1.0–64.3)
United Kingdom	16	47,526	11,846	9,498	1,321	12,965	461	809	10,626	86.7 (31.2–100)	13.3 (0–68.8)
Uzbekistan	1	105	35	3	0	0	0	0	67	NA	NA
**WHO European Region**	**417**	**649,620**	**114,056**	**244,671**	**12,300**	**149,339**	**5,005**	**10,620**	**113,629**	**82.6%**	**17.4%**

NA: not applicable (countries with three or fewer seasons with data); WHO: World Health Organization.

^a^ Only seasons with 100 or more reported influenza cases were included.

^b^ Not reported if there were fewer than five seasons of data.

### Spatiotemporal patterns of influenza epidemics

**Figure 2:**
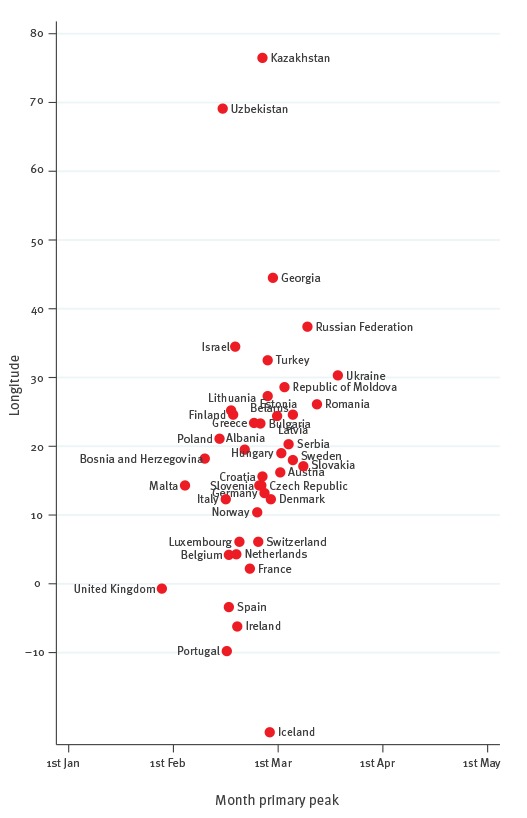
Timing of primary peak of influenza epidemics in the WHO European Region, by country longitude, WHO FluNet database, 2010–15

**Figure 3:**
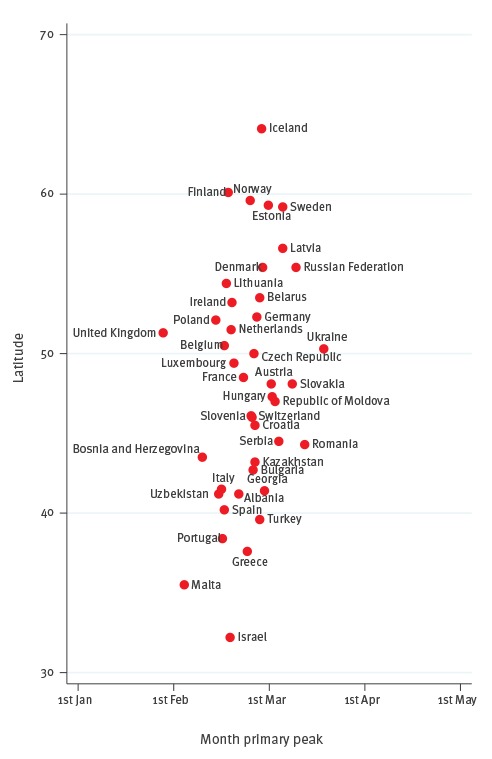
Timing of primary peak of influenza epidemics in the WHO European Region, by country latitude, WHO FluNet database, 2010–15

Influenza surveillance data for the period between week 27/2010 and week 26/2015 included 278,773 influenza A samples (72.6%) and 105,233 influenza B samples (27.4%) from 192 seasons in 42 countries. The timing of primary peaks obtained from the influenza circulation series of each country relative to, respectively, their longitudes and latitudes is shown in Figure 2 and Figure 3.

WHO: World health Organization.

WHO: World health Organization.

We found a notable coincidence in peak times: all countries (except the UK) had their primary peaks in February and March. Influenza epidemics usually peaked at the end of January in the UK - earlier than in the remaining countries. There was a non-significant longitudinal gradient in the timing of the primary peak, with countries in the west peaking earlier than those in the east (the typical timing of the primary peak fell at the end of January in the UK, and in mid-March in Ukraine). The p value was 0.125 when regressing the timing of the primary peak against the country’s longitude; however, the gradient became statistically significant (p = 0.001) when ignoring Kazakhstan, Uzbekistan and Iceland, which behaved as highly influential points in the model because of their geographical position. There appeared to be a slight, non-significant (p = 0.100) latitudinal gradient as well, with southern countries peaking a bit earlier than those countries with progressively higher latitudes in the north (for instance, mid-February in Spain and early March in Sweden). The time period between the earliest and latest country-specific influenza peaks was two months in the WHO European Region (Figure 2). Considering that influenza viruses circulate for two weeks before and after the peak is reached in any given country [21], it appears that a typical influenza season lasts an average of three months in the WHO European Region. Influenza A peaked earlier than influenza B in most countries (Table 2): the average time period between the peak of influenza A and B was 1.6 weeks.

**Table 2:** Timing of primary peak of influenza A and B epidemics in the WHO European Region, WHO FluNet database, 2010–15 (n = 384,006)

Country	Latitude	Longitude	Month primary peak		
			Influenza (any type)	Influenza A	Influenza B
Albania	41.1	20.3	2.68	2.66	2.96
Austria	47.6	14.3	3.02	2.88	3.47
Belarus	53.5	28.2	2.90	2.87	3.06
Belgium	50.7	4.8	2.53	2.58	2.42
Bulgaria	42.8	25.4	2.83	2.69	3.16
Bosnia and Herzegovina	44.2	18.0	2.30	2.29	3.48
Croatia	45.1	16.6	2.85	2.78	3.65
Czech Republic	49.8	15.5	2.84	2.62	3.67
Denmark	56.1	10.0	2.93	2.83	3.22
Estonia	58.6	26.0	2.99	2.95	3.19
Finland	64.4	26.5	2.57	2.55	2.66
France	46.5	2.7	2.73	2.71	2.78
Georgia	42.2	43.8	2.95	2.81	3.20
Germany	51.1	10.6	2.87	2.67	3.46
Greece	39.2	22.9	2.77	2.71	3.26
Hungary	47.2	19.6	3.03	2.87	3.59
Iceland	64.9	-18.3	2.92	2.77	3.47
Ireland	53.2	-8.0	2.61	2.69	2.33
Israel	31.4	35.2	2.59	2.54	2.92
Italy	42.9	12.3	2.50	2.42	2.86
Kazakhstan	48.2	67.6	2.85	2.78	3.08
Latvia	56.8	25.2	3.14	2.95	3.53
Lithuania	55.3	24.1	2.55	2.42	3.19
Luxembourg	49.8	6.3	2.63	2.64	2.61
Malta	35.5	14.3	2.11	2.00	2.76
Republic of Moldova	47.2	28.7	3.06	3.03	3.12
The Netherlands	52.2	5.8	2.60	2.44	3.29
Norway	67.5	15.8	2.80	2.77	2.87
Poland	52.1	19.6	2.44	2.30	3.44
Portugal	39.7	-7.8	2.51	2.52	2.48
Romania	45.9	25.2	3.37	3.27	3.53
Russian Federation	61.7	96.9	3.28	3.22	3.54
Serbia	44.0	21.0	3.10	3.02	3.46
Slovakia	48.8	19.8	3.24	3.15	3.47
Slovenia	46.1	15.0	2.82	2.65	3.34
Spain	40.4	-3.4	2.53	2.43	2.82
Sweden	62.8	16.9	3.14	3.00	3.56
Switzerland	46.8	8.4	2.81	2.72	3.06
Turkey	39.0	35.4	2.90	2.45	3.64
Ukraine	49.1	31.6	3.57	3.75	1.72
United Kingdom	53.9	-2.6	1.89	1.93	1.83
Uzbekistan	41.8	63.5	2.47	2.78	2.27

WHO: World Health Organization.

### Influenza transmission zones

For the cluster analysis, we included 290,915 influenza cases reported from July 2011 to June 2015 in 37 countries in the WHO European Region (all those included in the previous analyses except Bosnia and Herzegovina, Czech Republic, Kyrgyzstan, Malta, Slovakia and Uzbekistan).

The output of models with a five-cluster setting was largely inconsistent both between models and with respect to the ITZs proposed by the WHO. Results were highly dependent on the methodology used to derive the clusters and the parameters inputted into the model. Upon calculating the proportion of agreement and applying the algorithm described above, it was possible to identify a single ITZ, which included only seven countries (Austria, Denmark, Estonia, Georgia, Greece, Hungary and Republic of Moldova) that were largely non-contiguous with each other.

**Figure 4:**
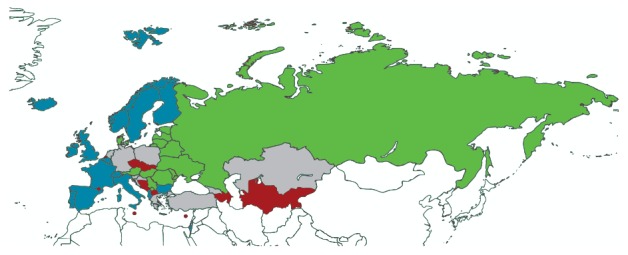
Partitioning of countries of the WHO European Region into two cluster models-derived influenza transmission zones, WHO FluNet database, July 2011–June 2015

The models’ outputs became progressively more consistent between one another when the number of clusters was reduced to four and three, although the ITZs were still small and not entirely sensible from a geographical standpoint as they were partly formed by non-neighbouring countries. Models assuming two clusters led to the identification of two data-driven ITZs which we have named ‘Western Europe’ and ‘Eastern Europe’ (Figure 4), although these labels were to some extent inaccurate: Albania, Bulgaria and Israel were assigned to the Western Europe ITZ and Denmark was assigned to Eastern Europe. The non-core countries were Ireland, Norway and the UK in the Western ITZ, and Estonia and Ukraine in the Eastern ITZ. The assignment of Greece and Poland to the ‘Eastern Europe’ ITZ, and of Slovenia to the ‘Western Europe’ ITZ, was not possible because their proportion of agreement with one country in the other ITZ was ≥ 70%. For the other non-assigned countries (Croatia, Georgia, Germany, Kazakhstan, Netherlands and Turkey), the inclusion and exclusion criteria were not met in two or more cases. 

WHO: World health Organization.

Blue: ‘Western’ zone; green: ‘Eastern’ zone; grey: countries not assigned to any influenza transmission zone; red: countries not included in the analysis.

Influenza epidemics started and peaked 2–3 weeks earlier in the Western than in the Eastern Europe ITZ (median week of start: 2 vs 4; median week of peak: 5 vs 8). There were no statistically significant differences in the median percentage of influenza cases that were caused by each virus (sub)type in countries belonging to the two ITZs (data not shown). Nine countries could not be assigned to either ITZ, some of which (the Netherlands, Germany, Slovenia, Croatia, Greece and Turkey) form a border or line between the two zones in a direction from the north-west to the south-east. Ireland in Western Europe, Georgia in the Caucasus region, Kazakhstan in Central Asia, and Poland were also non-classified countries.

## Discussion

We investigated the epidemiology and spatiotemporal patterns of influenza in the WHO European Region and evaluated whether the allocation of countries of this large world region into five ITZs (as proposed by WHO) could be confirmed from an epidemiological standpoint. Influenza A(H3N2) was most frequently the dominant virus in the study period (2000–15), followed by influenza A(H1N1) and influenza B. Epidemic peaks were distributed over a period of two months, with longitudinal (west-to-east) and latitudinal (south-to-north) gradients of timing. The peak of influenza B epidemics typically occurred later than those for influenza A, in agreement with earlier findings [22-25]. On the basis of our analysis, partitioning the WHO European Region into two ITZs (‘Western Europe’ and ‘Eastern Europe’) appeared to be the most appropriate, despite some inconsistencies in some countries in central Europe and along the border between the WHO European Region and Asia.

The introduction of the 2009 pandemic strain caused a change in the amount of data collected each season, which surged in 2009 and was then maintained at much higher levels compared with pre-pandemic times. The proportion of all available data collected in the seasons 1999–2008, 2009/10 and 2010–15 was 17.1%, 24.1% and 58.8%, respectively. The 2009 pandemic was a powerful incitement to ensure that many countries in the Region started to contribute their influenza surveillance data on a regular basis into a publicly accessible database (the WHO FluNet), which represents a valuable tool for researchers worldwide. Despite these important achievements, there are still some shortcomings in the quality and availability of influenza surveillance data in the WHO European Region. For instance, data are available for only a few countries on the Balkan peninsula, the Caucasus and central Asia, the proportion of influenza B cases that are being characterised is still very low, it would be desirable to have regional data for the Russian Federation, given its large population spread over a very vast territory, and, in contrast to the original EISS database [10], we noticed that the FluNet database has no data for a number of countries and seasons (e.g. Belgium 2005–07, Ireland 2005–07, the Netherlands 2003–07 and Slovakia 2003–07).

The study period covered by our analyses was characterised by the 2009 influenza pandemic, the first pandemic since 1968. Although influenza cases caused by the influenza A(H1N1)pdm09 virus strain were the most represented in the study database overall (probably because of the intense sampling effort during the 2009 pandemic), the majority of seasons were dominated by the A(H3N2) virus subtype, both before and after the pandemic year. Moreover, it is worth noting that the influenza B virus type caused a substantial proportion of influenza cases, as it accounted for a median of 17.2% of all cases reported in a season. These findings are in line with those of previous reports focusing on countries in both hemispheres and in the intertropical convergence zone [22,26] and highlight the role of influenza B as an important contributor to the total burden of disease of influenza.

We investigated the seasonal patterns of influenza circulation across a large range of latitudes and longitudes in Europe and part of western Asia. Because all countries are in the temperate region of the northern hemisphere, they all share the same winter timing and their seasonal patterns of influenza circulation were similar. The differences in the timing of influenza epidemics appeared to be smoothly distributed along a continuum in this large world area, without any clean break between countries or group of countries. The overall period of influenza activity can be estimated at about three months in a typical season, and there were longitudinal (west-to-east, significant) and latitudinal (south-to-north, not significant) patterns in the timing of seasonal peaks. Our findings suggest that the WHO European Region is not homogeneous with regard to the spread of influenza epidemics, though probably not so fragmented as to justify its partitioning into five ITZs.

Our analysis, based on data-driven clustering techniques, indicated that most countries of what is traditionally defined as Europe (i.e. the countries situated east of the Ural Mountains, not including Asian countries) can be grouped into two ITZs, separated by a curved line running across continental Europe in the direction from the north-west to the south-east. Compared with the partitioning proposed by WHO, most countries in the Northern Europe and South West Europe ITZs would merge into a single data-driven ITZ (which we named ‘Western Europe’; exceptions are Denmark, Germany, the Netherlands, Austria, Slovenia, Croatia, Serbia and Greece). The WHO-defined and data-driven ITZs for ‘Eastern Europe’ overlap well, except for the Baltic countries, Poland and Bulgaria. Finally, our results support the attribution of Turkey, the countries of the Caucasus and those in central Asia to extra-European ITZs, as proposed by the WHO; however, the limited availability of data for these countries does not allow definitive conclusions. The two data-driven ITZs differ from one another in the timing of epidemics but not in terms of circulating virus (sub)types, therefore the term ‘influenza transmission zone’ does not appear to be entirely appropriate and might be reconsidered.

We believe that establishing data-driven ITZs in the WHO European Region has important public health implications and can serve multiple purposes. Information on the course of influenza seasons could be developed and communicated at ITZ level in addition to the national level. Preparedness planning of seasonal influenza activity could be coordinated among countries included in the same ITZ. Also, the distribution of sentinel sites on the territory of countries within each ITZ could be redesigned so as to optimise the influenza surveillance activities in the ITZ as a whole. Because of the west-to-east and south-to-north gradients of spread, it may be worthwhile to evaluate whether, and to what extent, countries in the south-west of Europe could serve as sentinel sites for the rest of the WHO European Region (or at least for the Western Europe ITZ). Finally, by merging countries with similar patterns of influenza transmission, and in particular, with synchronised timing of influenza epidemics, the ITZs could be also seen as vaccination zones [27], i.e. groups of countries for which the timing of influenza vaccination campaigns could benefit from harmonisation.

A major strength of our study is the use of surveillance data from most countries in the WHO European Region for several consecutive influenza seasons. We used a range of complementary statistical techniques to study spatiotemporal patterns of influenza epidemics. As far as we know, this is the first study to assess the validity of the WHO-defined ITZs in a defined world Region of WHO. We chose to average the outputs from cluster analysis models with varying model specifications to increase the robustness of our results. However, because of the exploratory nature of our analytical approach, the limited number of seasons included in the cluster analysis, and some inconsistencies in the results, further analyses using alternative methods are warranted to confirm or refute our findings. For instance, this is the first study which has tried to define ITZs by averaging models from multiple clustering techniques, and we had no guidance on what thresholds we should use to define an ITZ. Also, different definitions to determine the start of an influenza season are available [28] and these may lead to different results. In addition, taking into account other parameters of an influenza season may help improve the partition of the WHO European Region into different ITZs.

We recommend that our cluster analysis for the WHO European Region is repeated within 3–4 years (with twice the amount of data) and the investigation is extended to bordering Regions. By including, for instance, Northern Africa and the Middle East, one may be able to categorise countries such as Turkey and Georgia that were not assigned to an ITZ in our analysis. This will allow a better definition of the ITZs for the WHO European Region and world-wide.
